# A long-term study indicates that tree clearance negatively affects fledgling recruitment to the Blue-fronted Amazon (*Amazona aestiva*) population

**DOI:** 10.1371/journal.pone.0267355

**Published:** 2022-06-01

**Authors:** Gláucia Helena Fernandes Seixas, Guilherme Mourão

**Affiliations:** 1 Parque das Aves, Foz do Iguaçu, Paraná, Brazil; 2 Fundação Neotrópica do Brasil, Bonito, Mato Grosso do Sul, Brazil; 3 Embrapa Pantanal, Corumbá, Mato Grosso do Sul, Brazil; MARE – Marine and Environmental Sciences Centre, PORTUGAL

## Abstract

The Blue-fronted Amazon (*Amazona aestiva*) is a Neotropical parrot with a large distribution in South America, including areas in Brazil, Bolivia, Argentina and Paraguay. A substantial part of the population of this parrot is concentrated in the Pantanal, a large wetland located in the center of South America. There, the clearing of forest and savannas has occurred through the years to accommodate beef-cattle pasture, and crops. Our objective was to understand the direct and indirect effects of time, availability of forest and savannas, and rainfall over the number of nestlings and fledged young of Blue-fronted Amazons inhabiting the southern Pantanal. We surveyed their nests from 1997 to 2018 and counted the number of nestlings produced and the number of fledglings capable to leave the nest in each year. Additionally, we used available data on the area covered by arboreal vegetation, and rainfall data collected at a Meteorological Station located in a central area of the study, as predictors of the numbers of nestlings and fledglings produced every year. Then, we applied structural equation modeling to examine both the direct and indirect effects of time, arboreal habitat availability, and annual cumulative rainfall on the number of nestlings and fledgling per nest. Finally, we estimated the long-term trend of the number of fledglings per nest as a surrogate to the populational trend of Blue-fronted Amazon. We found that none of the variables in our model explained the number of nestlings in nests, but the number of nestlings and the arboreal habitat availability directly and positively affected the number of fledglings. Time indirectly and negatively affected the number of fledglings per nest, whereas rainfall affected it indirectly positively. Additionally, we detected a concerning decrease of about 30% of the fledglings during the 22-years study, which could lead to a change in the species conservational status.

## Introduction

Parrots are the most threatened group of birds in the world [1]. Brazil concentrates the highest richness of psittacid species, with 69 species [2], of which 22 occur in the Pantanal wetlands [3]. The ecology and population trends of parrots are still poorly known [4–7], and often, the size of the distribution of species has been used as a proxy to establish its population status [1]. However, in some cases, relying only on the size of the distribution of a given species can obscure decreasing populational trends, especially in scenarios of widespread and rapid habitat destruction and/or climatic changes.

The Blue-fronted Amazon (*Amazona aestiva*) is a Neotropical parrot with a large distribution, occupying portions of the eastern, central and southern regions of South America, including areas in Brazil, Bolivia, Argentina and Paraguay [8]. Despite this large distribution, some authorities have stated that this species is now threatened [1,9] by a number of factors, such as illegal capture of nestlings to supply the pet trade, reduction of suitable habitats, forest fires and climatic changes [1,10–12], but none of these factors have been properly examined by long-term studies. A few years ago, the population status of the species was changed from “Least of Concern” (LC) to “Near Threatened” (NT) both by the International Union for Conservation of Nature—IUCN [1] and the Brazilian environmental authority–MMA [9].

Nesting cavities seems to be not limiting in Neotropical well-preserved humid forests [13]. However, a reduction in their availability is suspected to negatively impact the populations of the cavity-nesting Blue-fronted Amazon in dry-forested habitats, especially those subjected to selective logging [14]. Additionally, the quality of available cavities is related to the probability of nest success [15]. Thus, the availability of suitable cavities in number sufficient to arbor large populations of Blue-fronted Amazon, depends on the existence of large well-preserved areas covered by arboreal habitats as forests and savannas. The breeding activity of parrots involves several steps, including searching for a suitable cavity for nesting, laying eggs, incubating and raising nestlings, and they demand elevated energetic investment from the parents [16]. Therefore, the number of offspring produced in a given breeding season is not limited by the number of eggs laid, but by the number of nestlings that the parents can support until they are capable of leaving the nest [16]. Leaves, flowers, fruits and nectar are food items for adult parrots [17–19]. Thus, it is expected that changing in the area covered by arboreal vegetation would affect parrot’s productivity, by negatively impacting the availability of suitable cavities and food sources over the time. Additionally, in the Neotropical region, the productivity of parrots can be influenced by rainfall [20], which has a direct effect on the production of leaves and the processes of flowering and fruiting [21–24]. Understanding the relationship of parrots with their habitats and food sources allows us to determine which are their requirements and what resources and habitats need to be maintained to provide effective conservation [25].

Our objectives were to study the direct and indirect effects of time, availability of areas covered by forest and savannas, and rainfall over the number of nestlings and fledglings of Blue-fronted Amazon in a relatively long-term period (i.e., 22 years). We hypothesized that the change in time of the availability of arboreal habitats (i.e., including forest and savanna) would affect both the nestling number and the number of fledged parrots leaving the nest. Additionally, we expected that annual rainfall would affect the annual food supply for adult parrots, likely modulating parental investment in reproduction, affecting the size of the broods (i.e., number of nestlings in the nest) and the number of fledglings. Finally, we examined the long-term numbers of fledglings per nest per year, as it is directly related to populational recruitment.

## Materials and methods

### Ethics statement

This research did not demand capture or manipulation of the parrots. Licenses granted from the environmental Federal authority Ministério do Meio Ambiente/MMA allowing the conduction of this research were 12130–1; 12130–2; 324650; 43876–1; 43876–2; 43876–3.

### Data collection

**Fig 1:**
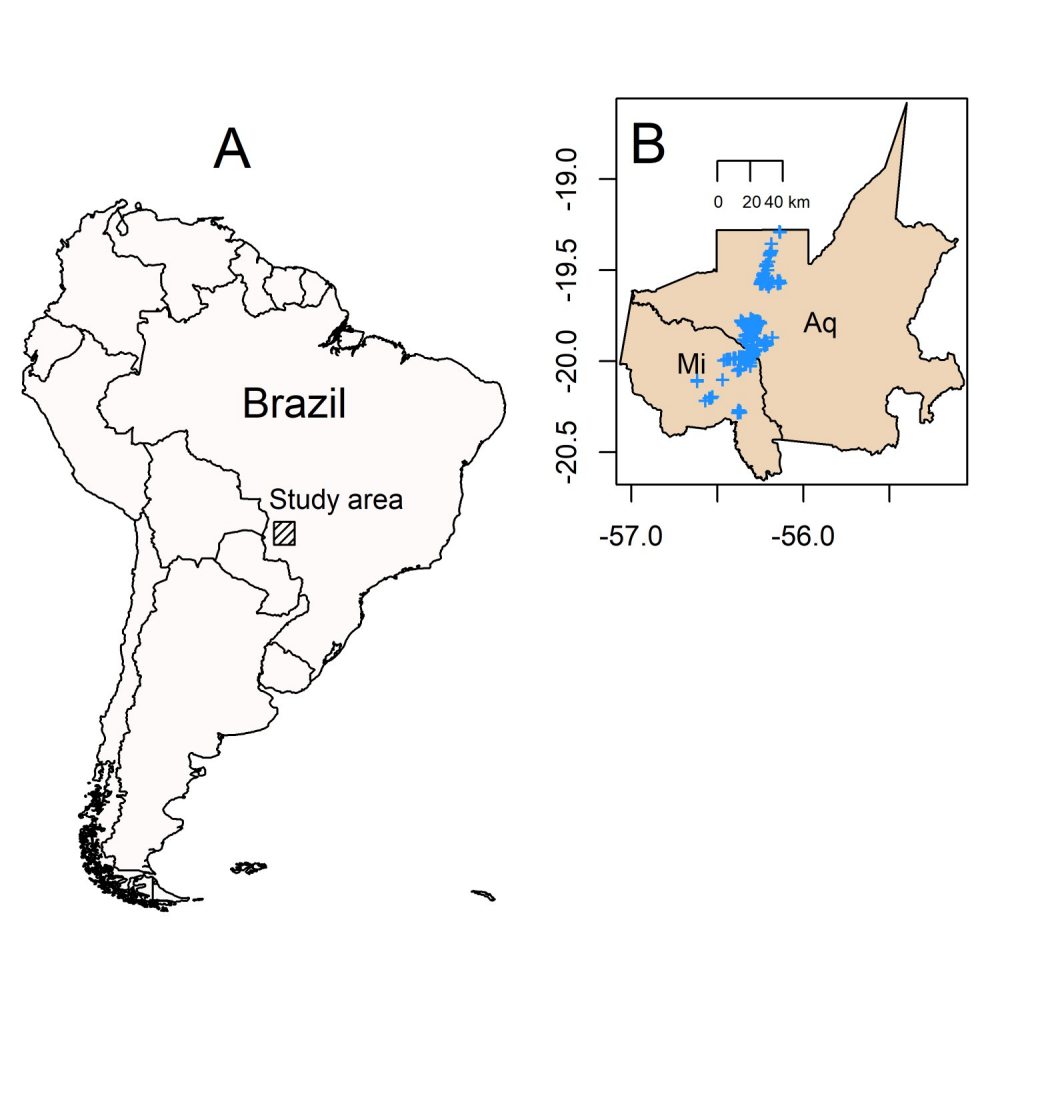
(a) Map of South America showing the location of the study area (hachured square), which included (b) two municipalities in Mato Grosso do Sul state in Brazil (Aq = Aquidauana and Mi = Miranda). Nests, shown as blue crosses, were monitored from 1997 to 2018 in a large area in the two municipalities.

We surveyed the nests of the Blue-fronted Amazon from 1997 to 2018 in a large area of the Aquidauana and Miranda municipalities of Mato Grosso do Sul, Brazil (Fig 1). Most of the area in these municipalities are occupied by the Pantanal, a large Neotropical wetland that covers approximately 179,300 km^2^ of Brazil, Bolivia and Paraguay [26]. The climate is marked by a rainy (November-April) and a dry season (May-October), and the mean annual rainfall is approximately 1200 mm [27]; however, a reduction on the mean annual rainfall has been observed in recent years, with an increase on the number of days without precipitation over the last 10 years, especially during the dry season [26].

As in most of the Pantanal, the landscape is characterized by a mosaic of semideciduous forests, savannas and flooded grasslands interspersed with forest patches. Beef cattle is the main economic activity, and the cattle used to graze almost exclusively on native grasses in large ranches. However, the areas covered by human made pastures in these ranches had increased during the last decades. In addition to these low terrain characteristics, in the southern portions of both municipalities, higher areas can be found covered with pastures, crops and patches of natural forests. This habitat heterogeneity allied to climatic seasonality creates a patchy distribution of flowers and fruits in space and time [28], leading parrots to roam in large areas around their nests in the search for food [24].

We spent an average of 29.9 days/year (10–54 days/year) on surveys, totaling 658 days of field work over the study period (1997–2018). From a 4x4 vehicle moving at low speed (i.e., ≤ 40km/h), we searched for nests as we crossed the internal unpaved roads of up to six ranches ranging from 6.5 km^2^ to 520 km^2^. Besides beef cattle, other economic activities in those ranches include eco-tourism and, more recently, irrigated rice in some of these ranches. In total, we surveyed an area of approximately 1960 km^2^, or an average of 441 km^2^ per year (SD = 247 km^2^). These surveys were conducted between July and December every year, when the parrots were nesting in that region. We concentrated our search efforts during the early morning and late afternoon, when the parrots were more active. In each year, we searched for new nest cavities and inspected the previously known nest cavities, and we were often informed by locals about nest locations. A multiple regression model indicates that the number of monitored nests increased linearly with the search effort, measured as survey days/year (β = 0.751, p_partial_ < 0.001), and with year (β = 0.602, p_partial_ = 0.027, F_2,19_ = 18.94, R^2^ = 0.67), presumably because the parental parrots tend to reuse the cavities previously used to nest, and many of them were becoming known by the researchers over time. Each nest cavity was inspected three to four times in a given breeding season, the last examination occurred when we expected that the nestlings were already fledged and almost ready to leave the nest. If fledglings were present and in good shape at this time, we assumed that they would successfully leave the nest. Details of the nest search and monitoring method are found in Seixas and Mourão [4]. When it was not possible to directly inspect the nest cavity, we used a compact digital camera (Canon Power Shot GLPH 135) to register the presence, number and condition of eggs, nestlings and fledglings within the nest cavity. The raw data are supplied (S1 Table).

The change from a given year to the next year in the absolute area covered by arboreal vegetation, including forest and savannas (Cerrado), was obtained by assessing the MapBiomas project [29], which is a consortium of institutions and nongovernmental organizations (NGOs) responsible for preparing and making available maps of vegetation change for every municipality of Brazil from 1985 to the present. The detailed methodology employed by the MapBiomas project has previously been published [29,30]. Daily rainfall data have been collected at the Meteorological Station of the Refúgio Ecológico Caiman ranch (19° 57’ 02” S, 56° 18’ 21” W) since 1972, which is in an approximately central area of this study, and they have kindly made available to us by the ranch administration.

We calculated the annual rainfall as the sum of monthly rainfall, considering the hydrological year to start in November of a previous year and to finish in October of the given year. This is convenient, especially because most of the nesting period of the Blue-fronted Amazon occurs during July-October, sometimes lasting until early December [31].

### Data analysis

Structural equation modeling (SEM) allows one variable to serve as a response in one path and as a predictor in another, and therefore, it is useful for quantify indirect effects that would be unrecognized by any single model [32,33]. For this reason, we applied SEM to examine both the direct and indirect effects of time (years), arboreal habitat availability, and annual cumulative rainfall on the number of nestlings and fledgling parrots per nest. For convenience, we used the function psem() available in the piecewiseSEM package [32] in the R environment for statistical analyses [34]. The piecewise structural equation models were made on a list of generalized least squares linear models, which represented our hypotheses (Table 1). The data were standardized and centered to allow a comparison between the parameters. We controlled the temporal autocorrelation of the explanatory variables “year” and “area covered by arboreal habitats” using a first-order autoregressive autocorrelation term available in the nlme package (function corCAR1) [35].

**Table 1:** Generalized least squares linear models (gls) listed in piecewise structural equation modeling (psem) were used to examine the direct and indirect effects of area covered by arboreal habitats, mean number of parrot nestlings present in nests and annual cumulative rainfall on the mean number of fledglings parrots per nest.

	gls Models
(1)	Fledgling parrots per nests ~ area covered by arboreal habitats + nestlings per nests + cumulative rain
(2)	Nestlings per nests ~ area covered by arboreal habitats + cumulative rain
(3)	Area covered by arboreal habitats ~ year + cumulative rain
(4)	Cumulative rain ~ year

Finally, to assess recruitment trends over time, we used a linear regression model relating the mean number of fledglings that flew per nest with time (years).

## Results

Over the twenty-two years of study, we registered 791 nests, 1067 nestlings and 628 fledglings. On average, we recorded 36 nests per year, ranging from 16 to 60 nests (S1 Table), with a mean of 48.5 nestlings per year (ranging from 21 to 83 nestlings per year). During this time, a mean of 28.5 fledged were recruited every year, ranging from 14 to 45 fledged per year, or an average of 0.8 (SD = 1) parrot fledged per nest each year.

During the study time, the area covered by arboreal vegetation generally decreased in the studied municipalities, which was not compensated by the recovery of tree-cover observed in some years (S1 Fig). It resulted in a loss of approximately 5980 km^2^ of arboreal vegetation (i.e., 26.7% of the total area of the municipalities), from the first to the last study year.

### Direct and indirect effects on fledged parrots per nest

**Fig 2:**
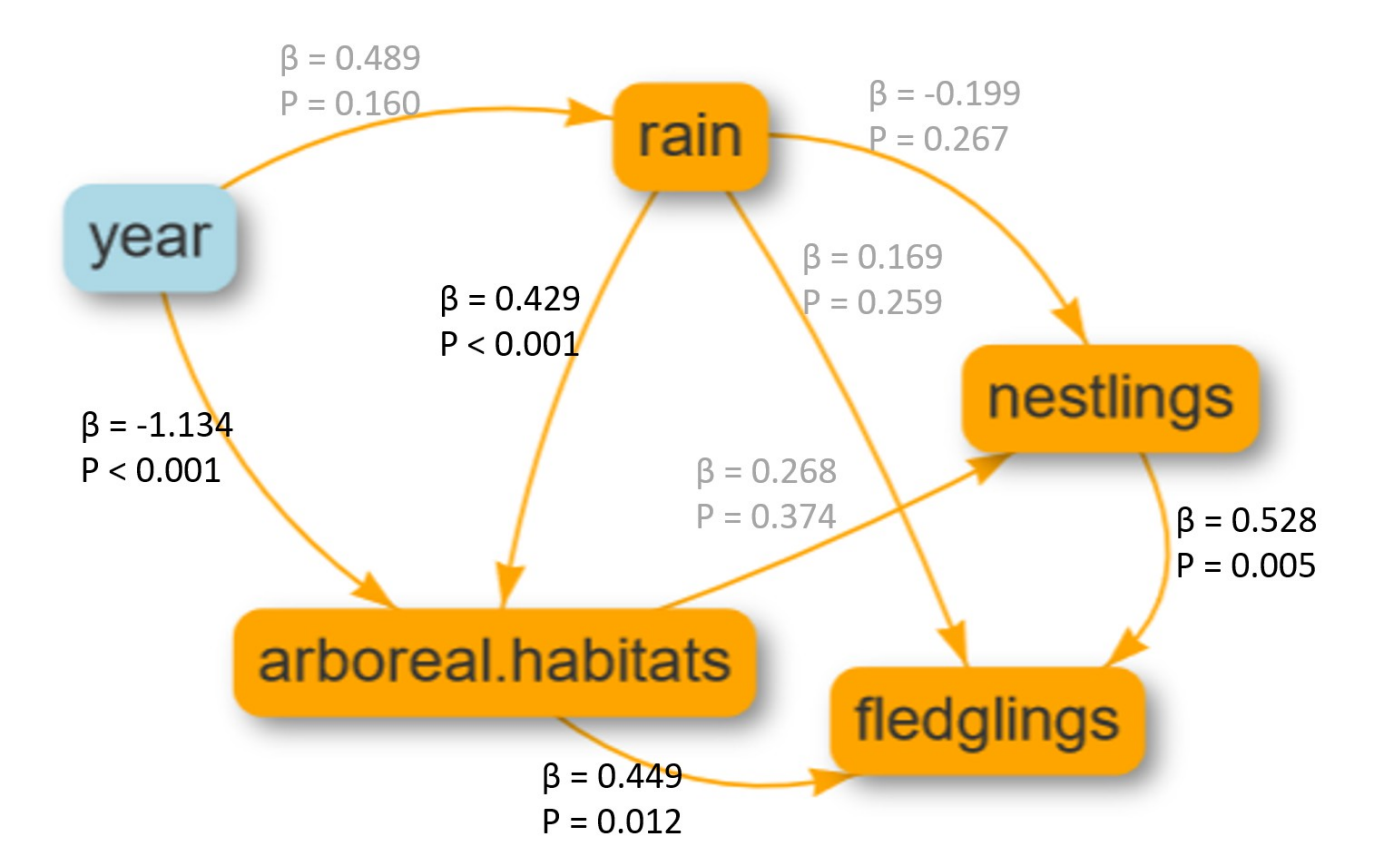
Diagram of the structural equation model constructed to explain the direct and indirect relationships between time (nesting year), annual cumulative rainfall measured in the study area, annual availability of arboreal habitats, which includes forest and savanna, number of nestlings of parrots per nest and number of fledgling parrots per nest. The study area comprised two municipalities in Mato Grosso do Sul state, Brazil (see Fig 1), and nests were monitored annually from 1997 to 2018. The standardized coefficients of the general least squares linear models (β) represent the relative strength of effects, and the probability is displayed for the significant effects (in black). Nonsignificant effects are noted in gray. Individual R^2^ values were 0.60 for the fledged response variable and 0.90 for arboreal habitats.

Both the area covered by arboreal habitats and the number of nestlings present per nest directly and positively affected the number of fledged parrots per nest (β = 0.449, p = 0.012 and β = 0.528, p = 0.005, respectively). The annual cumulative rainfall directly and positively affected the area covered by arboreal vegetation (β = 0.429, p < 0.001). For a given path line, the indirect effects are obtained by multiplying the coefficients of the direct effects along that line [32]. Thus, the annual cumulative rainfall indirectly contributed to the increase in fledged parrots per nest (β = 0.192). However, the direct effect of time (years) over the area covered by arboreal habitats was negative and larger in absolute value than these previous predictor factors (β = -1.131, p < 0.001). Therefore, the indirect effect of time on the number of fledged parrots per nest was negative (β = - 0.509). The structural equation model explained approximately 60% of the variability found in the number of fledged parrots per nest and 90% of the variability of the area covered by arboreal habitats every year, while the amount of explained variability in both the number of nestlings and annual rainfall was low and not significant (Fig 2).

### Long-term trend of the Blue-fronted Amazon population

With long-lived species such as the Blue-fronted Amazon, the population trend would be more accurately assessed examining the number of juveniles recruited to the adult population (i.e., number of fledged young that leave the nest) than by examining the number of breeding individuals. This occurs because in a population with recruitment problems, the number of breeding adults cannot vary much over the short-term until a large number of adults become senescent and incapable of breeding, and then the population could suffer a rapid decrease. Therefore, we examined the variation in the number of fledged parrots over time as a proxy for the populational trend. The mean number of fledged parrots per year decreased with time (β = -0.015, F_(1,20)_ = 5.187, p = 0.034, r^2^ = 0.21), indicating that populational recruitment suffered a decrease of approximately 30% during the 22 years of study.

## Discussion

Our structural equation modeling indicated that time (year) negatively affected the amount of arboreal habitat, including savannas and forests. Most of the clearance of arboreal vegetation in the Pantanal occurs to open space for cattle ranching, often replacing native vegetation with exotic grasses [36], although in some places in the study area, arboreal habitats give way to irrigated rice. The Blue-fronted Amazon depends on arboreal habitats for providing food, shelter, nest cavities [4,37], and nocturnal roosts [31]. Then, as the area covered by human made pastures and areas of irrigated rice cultivation increases in the Pantanal and surrounding areas, the habitat suitable for the Blue-fronted Amazon shrinks.

The model also indicated that cumulative rainfall over a twelve-month period was positively associated with the area covered with arboreal habitats. However, seedlings do not become adult trees in a twelve-month period, but in a large wetland, such as the Pantanal, the rate loss of arboreal vegetation tends to decrease in wet years because most areas are difficult to access for humans and their machines due to the floods. Additionally, deciduous and semideciduous trees tend to have larger crown areas in wet years than in dry years because they likely lose fewer leaves if water does not become limiting. In such a mosaic landscape as that in the study area, crowded with small forest patches, this effect should be sufficiently large to appear in the satellite images used by the MapBiomas project [29]. Regardless, trees with more leaves are prone to supply more food to the parrots, and the acquired energy can be used by the parents to successfully raise their brood [24]. Low nesting rates and reproductive success are known to occur in other parrots in disturbed habitats due to food limitation [4] or increases in predation rates of eggs and nestlings [38]. Many studies have reported a relationship between rain and flowering/fruiting for most plants in dry areas [19,23,24], which affects food availability and the reproductive success of female parrots [39].

None of the predictor variables of our model explained the number of nestlings per nest, although we expected that rain would directly affect it, as long-term studies indicated that changes in rainfall regimes can threatens populations of tropical birds [40], and reduction in annual rainfall was related to the reduction of clutch size in parrots [1]. On the other hand, the parrot response to eventual dry years may not be perceived in short time. For example, a severe drought event did not affect the clutch size of the Burrowing Parrots (*Cyanoliseus patagonus*) in Argentina, and the authors stated that the variation found in clutch size between seasons are more likely caused by differences in adult quality than by environmental conditions [41]. However, the lack of direct effect of rainfall over the number of parrots nestlings per nest may change in a near future. Some climate models project a reduction of about 30% of the rainfall in the Pantanal [26] as well as risk of occurrence of extreme droughts [42]. In this scenario, consecutive droughts likely will negatively impact the body conditions of the adults, determining clutch size reduction and decreasing the parental ability to raise their broods.

As expected, the number of nestlings was positively associated with the number of fledglings that successfully left the nest. Our results indicated that, throughout the study period, there was a decrease of approximately 1.5% in the number of fledglings that left the nest every year, which indicates a decrease of approximately 30% in the young entering the adult population during the 22 years of study.

Although poaching of nestlings for the local pet trade has been pointed out as the main factor threatening most Neotropical parrot populations [10], we have no direct evidence of severe capture of nestlings occurring in our study area. From 2004 to 2009, Seixas and Mourão [31] monthly counted the number of Blue-fronted Amazons in communal roosts approximately in the same area as of the present study, and observed a decrease of 1.1% in the number of young parrots recruited to the adult population per year. This figure is very similar to the present estimate, although it was obtained from a very different type of data, reinforcing the idea that another factor rather than poaching may be jeopardizing the population of Blue-fronted Amazons in the southern part of the Pantanal and surrounding areas. Our results indicate that the availability of arboreal habitats has a direct association on the number of fledged parrots that leave the nest every year and, therefore, the clearing of forest and savannas that has occurred through the years to accommodate human pasture and crop production jeopardizes populational recruitment. The clearing of woody vegetation can negatively affect parrots in different ways. This leads to the direct destruction of parrot cavities and broods within these cavities and to a reduction in food availability for parrots [14,31,37]. Clearing in the Pantanal is usually followed by fire, what further aggravates the factors mentioned above. Thus, parrots become more exposed to their predators as shelters become scarce [31]. Climatic changes models suggested a 5C to 7C increase in mean air temperature and a severe reduction in the rainfall for the Pantanal until 2100 [26,42]. Additionally, in recent years, the Pantanal has experienced extreme drought, mainly caused by reduced transport of warm and humid summer air from Amazonia into the Pantanal [26]. Historically, drought, fire and clearing have always occurred together in the Pantanal, but now the situation has become so critical that these authors stated that "If current climate and land-management trends persist, the Pantanal as we know it will cease to exist" [26].

Habitat loss, however, is not limited to the two municipalities studied here or even to the Pantanal as whole, but it is widespread in Brazil, especially in the Brazilian savanna (Cerrado). The Brazilian Cerrado is the largest savanna in South America, with approximately two million square kilometers, and most of the Blue-fronted Amazon distribution occurs within the Cerrado and Pantanal. Some authorities stated that the Cerrado is now the most threatened ecoregion in Brazil due to agricultural expansion [43]. From 1985 to 2017, approximately 247 thousands of square kilometers of native vegetation were lost, with an annual loss rate of 0.5% [43]. This indicates that the recruitment of young adults to the adult parrot population could be jeopardized in most of the Blue-fronted Amazon distribution. Our 22-year-long study revealed a serious negative effect of arboreal vegetation clearing in the recruitment of young parrots in the Pantanal, an area that is relatively free from the extraction of nestlings to supply the illegal pet trade. However, in many other areas in the parrot’s distribution, this threat is combined with that of poaching, increasing the threats to the population. For example, in the catchment area of the Paraná River, an area of Cerrado vegetation, our preliminary studies indicated that most nests are destroyed to allow poachers to reach nestlings (G. Seixas. [Unpublished]). Besides, there are reports of decrease of the populations of Blue-fronted Amazon inhabiting even remote areas in the Chaco in Argentina [14] and in the Brazilian Caatinga [44]. A few years ago, the IUCN and MMA changed the species status from less concern to near threatened. However, unless the environmental policies are changed in a very radical way to provide more protection to their arboreal habitats in Brazil, it is likely that the IUCN and MMA will need to change the Blue-fronted Amazon status once again to a more restrictive status soon.

## Supporting information

S1 Fig Evolution of arboreal habitat area (km^2^) in two municipalities in Mato Grosso do Sul state in Brazil (Aq = Aquidauana and Mi = Miranda) from 1997 to 2018 according to the MapBiomas database for 2018 [29,30]. The trendline and 95% CI bands were estimated by a generalized additive model using integrated smoothness using the mgcv package [45], and plotted using the visreg package [46].
(PDF)

S1 Table Counts of the number of eggs, nestlings and fledglings, and geographic coordinates of every nest surveyed from 1997 to 2018. (CSV)
