# Supplementary material for: A long-term study indicates that tree clearance negatively affects fledgling recruitment to the Blue-fronted Amazon (Amazona aestiva) population
Source: PLoS One. 2022 Jun 1;17(6):e0267355. doi: 10.1371/journal.pone.0267355 (PMC9159586; doi:10.1371/journal.pone.0267355)

**S2 Fig. Evolution of arboreal habitat area (km$^2$) in two municipalities in Mato Grosso do Sul state in Brazil (Aq = Aquidauana and Mi = Miranda) from 1997 to 2018 according to the MapBiomas database for 2018 [29, 30].** The trendline and 95% CI bands were estimated by a generalized additive model using integrated smoothness using the mgcv package [45], and plotted using the visreg package [46].

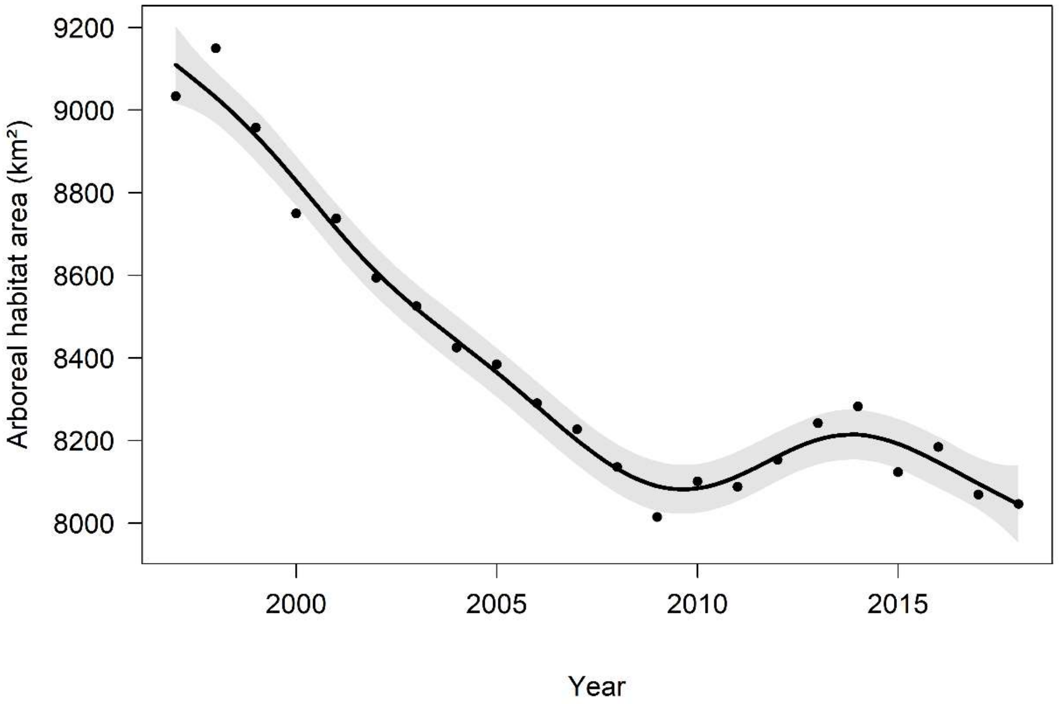

Supplement: S1 Fig — The trendline and 95% CI bands were estimated by a generalized additive model using integrated smoothness using the mgcv package [45], and plotted using the visreg package [46]. (PDF) [file pone.0267355.s001.pdf]
